# Efficient perovskite light-emitting diodes based on a solution-processed tin dioxide electron transport layer†
†Electronic supplementary information (ESI) available. See DOI: 10.1039/c8tc01871e


**DOI:** 10.1039/c8tc01871e

**Published:** 2018-06-06

**Authors:** Heyong Wang, Hongling Yu, Weidong Xu, Zhongcheng Yuan, Zhibo Yan, Chuanfei Wang, Xianjie Liu, Mats Fahlman, Jun-Ming Liu, Xiao-Ke Liu, Feng Gao

**Affiliations:** a Department of Physics , Chemistry and Biology (IFM) , Linköping University , Linköping 58183 , Sweden . Email: xiaoke.liu@liu.se ; Email: feng.gao@liu.se; b Laboratory of Solid State Microstructures , Innovation Center of Advanced Microstructures , Nanjing University , Nanjing 210093 , P. R. China; c State Key Laboratory of Luminescent Materials and Devices , South China University of Technology , Guangzhou 510640 , P. R. China; d State Key Lab of Silicon Materials , Zhejiang University , Hangzhou 310027 , P. R. China

## Abstract

To achieve high-performance perovskite light-emitting diodes (PeLEDs), an appropriate functional layer beneath the perovskite emissive layer is significantly important to modulate the morphology of the perovskite film and to facilitate charge injection and transport in the device. Herein, for the first time, we report efficient n–i–p structured PeLEDs using solution-processed SnO_2_ as an electron transport layer. Three-dimensional perovskites, such as CH(NH_2_)_2_PbI_3_ and CH_3_NH_3_PbI_3_, are found to be more chemically compatible with SnO_2_ than with commonly used ZnO. In addition, SnO_2_ shows good transparency, excellent morphology and suitable energy levels. These properties make SnO_2_ a promising candidate in both three- and low-dimensional PeLEDs, among which a high external quantum efficiency of 7.9% has been realized. Furthermore, interfacial materials that are widely used to improve the device performances of ZnO-based PeLEDs are also applied on SnO_2_-based PeLEDs and their effects have been systematically studied. In contrast to ZnO, SnO_2_ modified by these interfacial materials shows detrimental effects due to photoluminescence quenching.

## Abstract 2

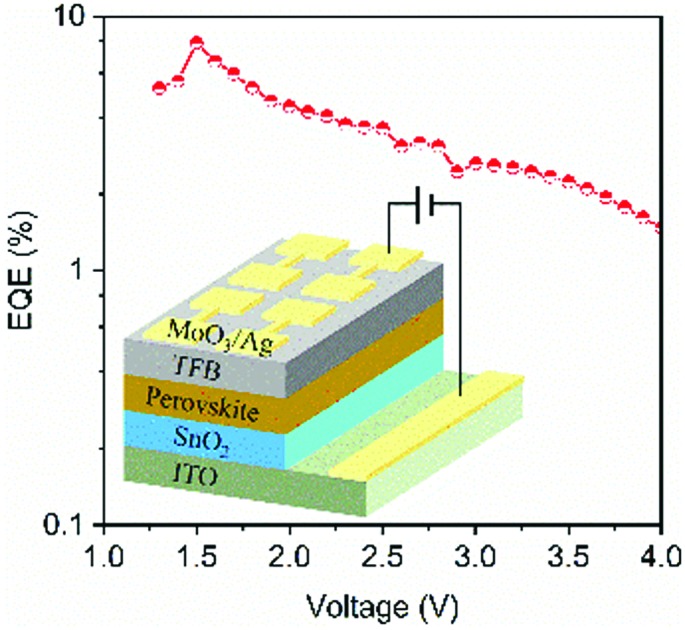
Solution-processed tin dioxide is employed as an electron transport layer in n–i–p-structured perovskite light-emitting diodes realizing an EQE of 7.9%.

## Introduction

Metal halide perovskites have attracted enormous attention in recent years as promising semiconducting materials in optoelectronic applications, such as solar cells, light-emitting diodes (LEDs), lasers, transistors and photodetectors.^1^–^5^ These materials show exceptional properties such as suitable bandgaps, high absorption coefficients, low trap densities, and long carrier diffusion lengths, leading to rapid progress in solar cells, among which a high power conversion efficiency (PCE) over 22% has been achieved.^6^,^7^ Furthermore, they also exhibit outstanding light-emitting properties, such as high colour purity, readily tuneable emissions and high photoluminescence quantum yields (PLQYs), making them promising candidates for displays and lighting applications.^8^,^9^ Within the last four years, increasing efforts have been devoted to developing efficient perovskite LEDs (PeLEDs).^10^–^17^

To realize high-efficiency PeLEDs, charge transport layers, including hole transport layers (HTLs) and electron transport layers (ETLs), are employed to balance hole- and electron-injection and transport.^18^ In general, there are two types of device architectures for PeLEDs, p–i–n and n–i–p, which refer to a layer preparation sequence in the form of a p-type layer, an intrinsic layer and an n-type layer and the reverse sequence, respectively. For efficient p–i–n based PeLEDs, vacuum-deposited organic molecules, such as 2,2′,2′′-(1,3,5-benzenetriyl)-tris(1-phenyl-1-*H*-benzimidazole) (TPBi) and 2,9-dimethyl-4,7-diphenyl-1,10-phenanthroline (BCP), are employed as the ETLs.^10^,^19^ In contrast, efficient n–i–p based PeLEDs are fabricated through all solution processes, where colloidal zinc oxide (ZnO) nanoparticles are usually used as the ETLs.^11^,^20^–^24^ Despite high efficiencies in PeLEDs, ZnO could cause the problem of chemical instability in the perovskite films,^25^,^26^ limiting its applications in PeLEDs. Therefore, it is desired to develop new types of solution-processed ETLs for efficient n–i–p based PeLEDs.

Solution-processed inorganic metal oxides, such as titanium dioxide (TiO_2_) and tin dioxide (SnO_2_), are potential ETL candidates for efficient PeLEDs. These materials have large bandgaps, a suitable conduction band minimum (CBM) for electron injection and a deep valence band maximum (VBM) for hole blocking.^27^–^29^ In addition, their thin films show good transparency in the visible-infrared region of 400–900 nm, minimizing optical energy losses caused by the ETL.^30^,^31^ Efforts have been made to develop PeLEDs using solution-processed TiO_2_, which however show a relatively low external quantum efficiency (EQE) of 0.48%.^14^ Moreover, preparation of the TiO_2_ layer requires high-temperature (>450 °C) sintering, which is a barrier for low-cost and stretchable PeLEDs. In addition to TiO_2_, solution-processed SnO_2_ has also been widely used as the ETL in organic, dye-sensitized, and perovskite solar cells.^28^–^33^ Furthermore, SnO_2_ has high mobilities (∼240 cm^2^ V^–1^ s^–1^ and 1.9 × 10^–3^ cm^2^ V^–1^ s^–1^ for bulk and nanoparticle films, respectively) and a high electrical conductivity (1.82 × 10^–8^ S cm^–1^ at room temperature).^34^,^35^ For instance, You and co-workers reported hysteresis-free perovskite solar cells with PCEs of around 20% based on colloidal SnO_2_ nanoparticles.^34^ Given that the SnO_2_ film has suitable energy levels, good transparency and high electron mobility, it is thus natural to expect that the use of the solution-processed SnO_2_ ETL would be a fruitful approach for obtaining high-efficiency solution-processed PeLEDs.

Herein, for the first time, we report efficient n–i–p structured PeLEDs using solution-processed SnO_2_ as the ETL. It is found that three-dimensional (3D) perovskites, such as CH(NH_2_)_2_PbI_3_ (FAPbI_3_) and CH_3_NH_3_PbI_3_ (MAPbI_3_), show considerably enhanced chemical compatibility with SnO_2_ compared with ZnO. More importantly, both 3D- and low-dimensional perovskite based PeLEDs using SnO_2_ as the ETL exhibit high efficiencies, among which a high EQE of 7.9% has been realized. These results suggest that SnO_2_ can be used as an efficient ETL in solution-processed PeLEDs. Furthermore, interfacial materials, such as polyethylenimine ethoxylated (PEIE) and polyethylenimine (PEI), that have been widely used to improve the device performances of ZnO-based PeLEDs are also employed in SnO_2_-based PeLEDs, the effects of which have been systematically studied. Unexpectedly, these two interfacial materials show detrimental effects on SnO_2_ based PeLEDs due to photoluminescence quenching.

## Results and discussion

**Fig. 1:**
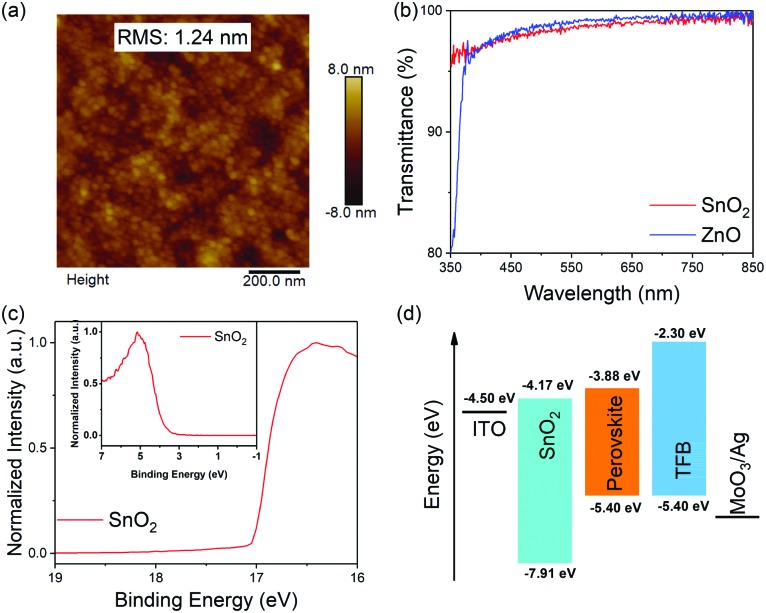
(a) AFM height image of the SnO_2_ film. (b) Transmission spectra of the SnO_2_ and ZnO films deposited on ITO-coated glass substrates, respectively. (c) UPS cutoff edge of the SnO_2_ film, inset: valence band edge. (d) Flat-band energy-level diagram of the materials used in this study.

The morphology of the SnO_2_ film obtained by spin-coating colloidal SnO_2_ nanoparticles on an ITO-coated glass substrate is investigated by using atomic force microscopy (AFM). As shown in Fig. 1a, a smooth and pinhole-free SnO_2_ film with a root-mean-square (RMS) roughness of 1.24 nm is obtained. Fig. 1b shows the transmission spectra of the SnO_2_ and ZnO films prepared on ITO-coated glass substrates, respectively. The SnO_2_ film exhibits similar transparency to the ZnO film between 420 and 850 nm. Note that the SnO_2_ film shows excellent transparency with transmission values greater than 97% in the ultraviolet-visible-infrared region, suggesting that the SnO_2_ layer will cause minimized optical energy losses in PeLEDs with a wide range of emissive wavelengths. In particular, in the ultraviolet region below 400 nm, where the ZnO film starts to absorb light (the bandgap of ZnO is ∼3.53 eV),^36^ the SnO_2_ film still shows good transparency with transmission values greater than 95% due to a much larger bandgap of ∼3.74 eV,^34^ showing promise as a good ETL candidate for ultraviolet LEDs.

The electronic states and band structures of the SnO_2_ film and a low-dimensional perovskite film consisting of FAPbI_3_: 60 mol% NMAI (NMAI = C_10_H_7_CH_2_NH_2_·HI) are studied by ultraviolet photoelectron spectroscopy (UPS). The work functions (WFs) of the SnO_2_ and perovskite films are calculated to be –4.17 eV and –4.18 eV, respectively (Fig. 1c and Fig. S1a, ESI†). The CBM and VBM of the perovskite film calculated from the UPS spectra and Tauc plot (Fig. S1b, ESI†) are –3.88 eV and –5.40 eV, respectively. As shown in Fig. 1d, the CBM of the SnO_2_ layer matches well with that of the perovskite layer, facilitating efficient electron injection from the ETL into the perovskite emissive layer; the valence band maximum of the SnO_2_ layer (–7.91 eV) is much deeper than that of the perovskite layer, offering efficient hole blocking. Therefore, the SnO_2_ film exhibits smooth morphology, excellent transparency and suitable energy levels, which is promising as an ETL candidate for PeLEDs.

**Fig. 2:**
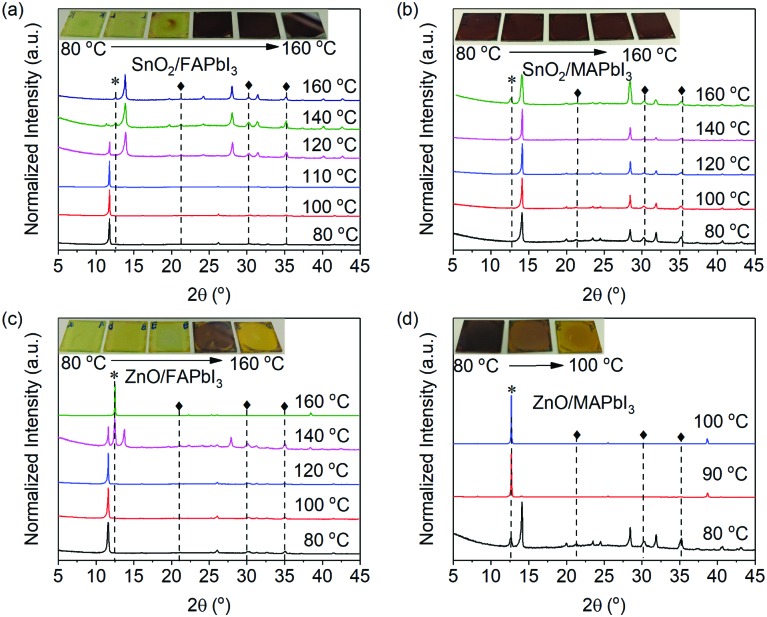
XRD patterns and photographs of 3D perovskite films deposited on the SnO_2_ and ZnO films with various annealing temperatures. (a) FAPbI_3_ films deposited on SnO_2_, (b) MAPbI_3_ films deposited on SnO_2_. (c) FAPbI_3_ films deposited on ZnO. (d) MAPbI_3_ films deposited on ZnO. * represents the diffraction peak of PbI_2_, ♦ represents the diffraction peak of ITO.

We use a one-step spin-coating method to form 3D perovskite films (FAPbI_3_ and MAPbI_3_) on the SnO_2_ and ZnO films with various annealing temperatures to study their chemical compatibility with the underlying SnO_2_ and ZnO, respectively. The crystal structures of these films are investigated by X-ray diffraction (XRD). As shown in Fig. 2a, the FAPbI_3_ films deposited on SnO_2_ show a dominant diffraction peak at 11.8° when they are annealed below 120 °C, which can be assigned to the (010) lattice plane of the yellow phase.^37^,^38^ Interestingly, a diffraction peak at 13.9° that belongs to the (111) lattice plane of the black phase can be observed when annealed at 120 °C, accompanied by the colour change of the perovskite films (Fig. 2a). When they are annealed above 120 °C, the FAPbI_3_ films start to decompose into lead iodide (PbI_2_), the (001) lattice plane of which is located at 12.6°. However, it is worth noting that the FAPbI_3_ film shows a pure black phase when annealed at 160 °C for 1 min although decomposition starts at longer annealing times (Fig. S2, ESI†). In comparison, the FAPbI_3_ films deposited on ZnO start to decompose into PbI_2_ when the annealing temperature is higher than 120 °C, below which the films are in the yellow phase. The colour of the films is an obvious indication showing the change of the phases (Fig. 2c). Similarly, the MAPbI_3_ films deposited on ZnO starts to decompose into PbI_2_ when annealed at 80 °C, and the diffraction peak of MAPbI_3_ is hardly observed when annealed at 90 °C and 100 °C, accompanied by an obvious colour change of the films (Fig. 2d). It is impressive that the MAPbI_3_ films deposited on SnO_2_ are stable below 100 °C; no obvious colour change of the perovskite films is observed at a high annealing temperature up to 160 °C (Fig. 2b). These results demonstrate that the 3D perovskite films show considerably enhanced chemical compatibility with SnO_2_ compared with ZnO, suggesting feasible applications of SnO_2_ in PeLEDs where perovskite films require a wide range of annealing temperatures.

**Fig. 3:**
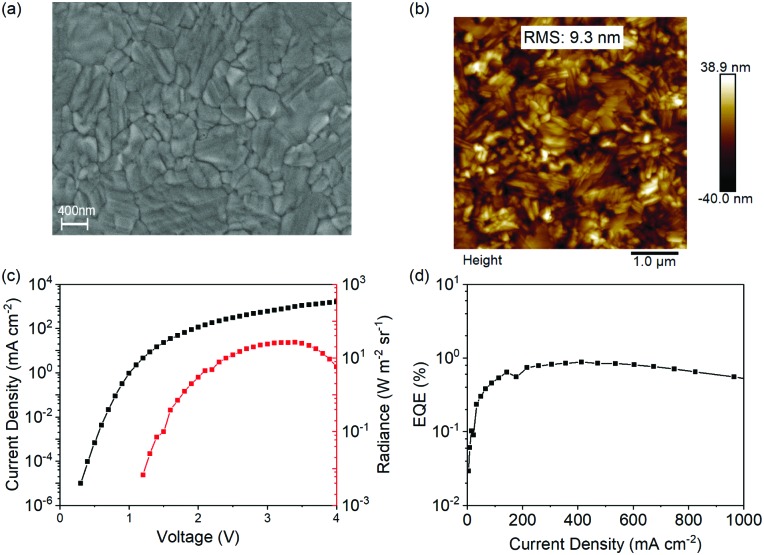
(a) SEM image and (b) AFM image of the FAPbI_3_ film deposited on SnO_2_. (c) Current density–voltage–radiance and (d) EQE–current density curves of the device based on FAPbI_3_ deposited on SnO_2_.

We further study the morphologies and optoelectronic properties of the FAPbI_3_ film (annealed at 120 °C) deposited on SnO_2_. As shown in Fig. 3a and b, the film shows smooth (RMS: 9.3 nm) and pinhole-free morphologies with large grains. The absorption and PL spectra of the FAPbI_3_ film deposited on SnO_2_ were also measured and are shown in Fig. S3 (ESI†). The absorption onset is located at 813 nm, corresponding to an energy bandgap of 1.52 eV. In addition, this film shows narrow emission peaked at 811 nm with a PLQY of 7%. A PeLED is fabricated with the structure of indium tin oxide (ITO)/SnO_2_ (30 nm)/FAPbI_3_ (480 nm)/poly(9,9-dioctyl-fluorene-*co-N*-(4-butylphenyl)diphenylamine) (TFB, 40 nm)/MoO_3_ (7 nm)/Ag (80 nm). As shown in Fig. 3c, the device has a very low turn-on voltage (the driving voltage when emission is detectable) of 1.2 V and a maximum radiance of 26 W sr^–1^ m^–2^. It is interesting to note that the turn-on voltage (*V*_on_) of this device is quite lower than the theoretical limit of 1.52 V according to the bandgap (*E*_g_) of FAPbI_3_ (*V*theoreticalon = *E*_g_/*e*, where *e* is the unit charge), which may be explained by an Auger-assisted energy upconversion model.^39^–^41^ Since TFB has a hole mobility of ∼1.0 × 10^–2^ cm^2^ V^–1^ s^–1^ which is an order of magnitude higher than that of the electron mobility of the SnO_2_ nanoparticle film, there is a negligible hole injection barrier between the perovskite layer and TFB.^42^ Thus, holes and electrons are likely to accumulate at the interface between SnO_2_ and the perovskite layer. With electrons accumulating at the interface, an Auger-assisted electron injection process can take place, in which one high-energy electron can be generated after absorbing the energy released from the interfacial recombination of an electron–hole pair. The resulting high-energy electron can overcome the injection barrier between the perovskite layer and SnO_2_ and recombine with the hole inside the perovskite EML to emit a photon. In addition, this device exhibits a very low EQE at low current density which increases to a moderate maximum value of 0.9% at a high current density of ∼400 mA cm^–2^. This finding can be explained by the low exciton binding energy of the large-grain FAPbI_3_ crystals, where non-radiative trap-assisted recombination is dominant at low carrier excitation densities and radiative bimolecular recombination gradually increases with increasing excitation density.^43^

**Fig. 4:**
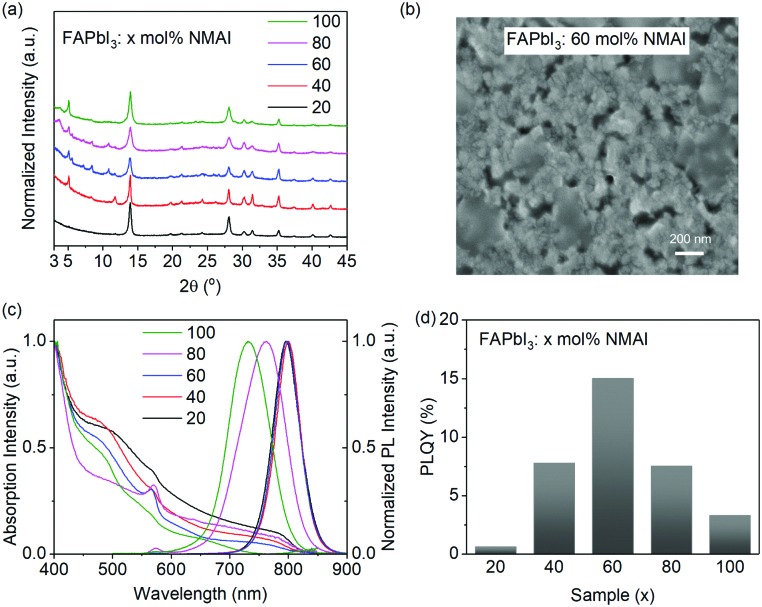
(a) XRD patterns, (b) top-view SEM image, (c) absorption and PL spectra, and (d) PLQYs of FAPbI_3_: *x* mol% NMAI (*x* = 20, 40, 60, 80, and 100) films prepared on SnO_2_.

In order to investigate the generality of the SnO_2_ ETL in PeLEDs, we further investigated low-dimensional perovskites prepared on SnO_2_. Low-dimensional perovskites can be synthesized by incorporating bulky organic ammonium cations that are too large to fit into the interspace between the PbX_6_^4–^ (X = I, Br, and Cl) octahedral layers and therefore split large perovskite grains into different types of nanocrystals.^20^,^43^,^44^ In this study, we prepare a series of low-dimensional perovskites by adjusting the molar ratios between 1-naphthylmethylammonium iodide (NMAI) and FAPbI_3_, expressed as FAPbI_3_: *x* mol% NMAI (*x* = 20, 40, 60, 80, and 100). These films are formed by one-step spin-coating from precursor solutions on SnO_2_, followed by thermal annealing at 100 °C. XRD measurements are conducted to determine the composition and crystallinity of the perovskite films. As mentioned above, the FAPbI_3_ film annealed at 100 °C is in the yellow phase (Fig. 2a), whereas the addition of NMAI lowers the transition temperature of the black phase (Fig. 4a). With 20 mol% NMAI, most of the perovskites have been transferred into the black phase. With further increasing ratios of NMAI, the diffraction peaks assigned to layered perovskites are observed, indicating that the as-formed films are composed of grains distinct from the 3D perovskites. The average crystal sizes of the low-dimensional perovskite films are calculated from the XRD results by using the Debye–Scherrer equation (eqn (S1), ESI†). As shown in Table S1 (ESI†), the addition of NMAI gradually decreases the average crystal size from 28.3 nm (3D FAPbI_3_) to 16.2 nm (*x* = 0.8). The top-view scanning electron microscopy (SEM) image of FAPbI_3_: 60 mol% NMAI clearly shows the formation of tiny grains (Fig. 4b). AFM measurements are also performed to investigate the morphology of this film. As shown in Fig. S4 (ESI†), the film consists of small grains and exhibits a RMS of 16.6 nm.

The normalized absorption and PL spectra of these low-dimensional perovskite films are shown in Fig. 4c. The addition of 20 and 40 mol% NMAI to FAPbI_3_ slightly changes the absorption and PL spectra, whereas more amounts of NMAI induce the formation of the slightly changes the absorption and PL spectra, whereas more amounts of NMAI induce the formation of the 〈*n* = 2 = 2〉-layered perovskite, in which the characteristic absorption peak is located at 573 nm.-layered perovskite, in which the characteristic absorption peak is located at 573 nm.^20^,^44^,^45^ The PL spectra, peaked at 798 nm with a full width at half maximum (FWHM) of 54 nm, almost remain the same when the NMAI contents are below 60 mol%. It is also interesting to note that obvious blue-shifted PL spectra are observed when 80 and 100 mol% NMAI are introduced (PL spectra peaked at 762 nm and 732 nm, respectively), which may be caused by the size-dependent quantum confinement effect.^46^ In addition, the FWHM of these two spectra are broadened to 89 nm (*x* = 80) and 81 nm (*x* = 100), respectively. The wider PL spectra may be originated from several low-dimensional emissive components – as indirect evidence of a clear PL peak at 573 nm that derives from the = 100), respectively. The wider PL spectra may be originated from several low-dimensional emissive components – as indirect evidence of a clear PL peak at 573 nm that derives from the 〈*n* = 2 = 2〉 -layered perovskite can be observed. The PLQYs of these films change along with the ratios of NMAI, among which the addition of 60 mol% NMAI contributes to the highest PLQY of 15% ( -layered perovskite can be observed. The PLQYs of these films change along with the ratios of NMAI, among which the addition of 60 mol% NMAI contributes to the highest PLQY of 15% (Fig. 4d).

**Fig. 5:**
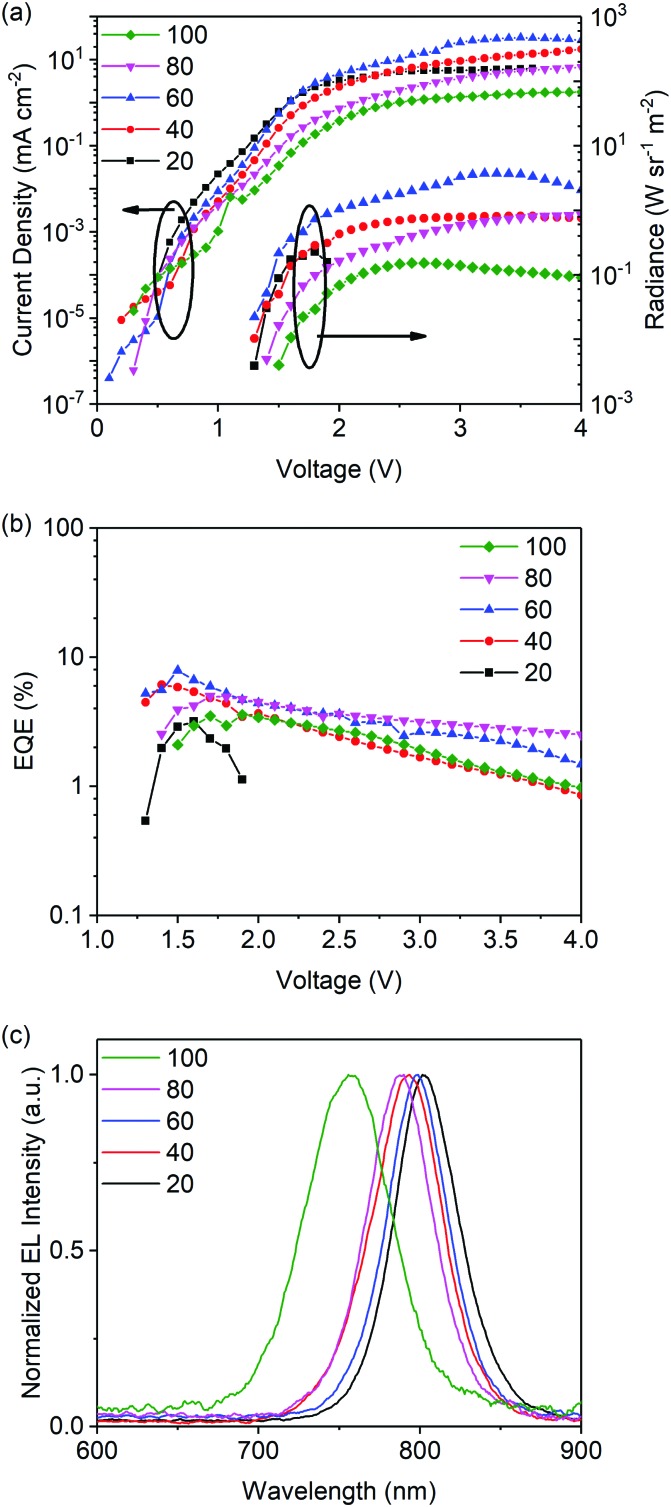
(a) Current density–voltage–radiance curves, (b) EQE–voltage curves, and (c) EL spectra of low-dimensional PeLEDs with FAPbI_3_: *x* mol% NMAI (*x* = 20, 40, 60, 80, and 100) films as the EMLs and SnO_2_ as the ETLs.

PeLEDs are fabricated to investigate the electroluminescent (EL) properties of these low-dimensional perovskites with SnO_2_ as the ETL. The device structure is the same as that of the FAPbI_3_-based PeLED except the emitting layer. The current density–voltage–radiance curves of these PeLEDs are shown in Fig. 5a. It should be noted that the addition of NMAI gradually decreases the current densities of the devices due to the insulating nature of NMAI. In addition, the FAPbI_3_: 80 mol% NMAI and FAPbI_3_: 100 mol% NMAI-based devices show gradually increased turn-on voltages compared with those with lower amounts of NMAI. This can be explained by their relatively wider bandgaps. The EQE–voltage curves of these devices are shown in Fig. 5b, among which the FAPbI_3_: 60 mol% NMAI based device shows the highest EQE of 7.9%. The statistical external quantum efficiency from 44 devices presents an average EQE of 6.8% (Fig. S5, ESI†). The results demonstrate that SnO_2_ is a good ETL for PeLEDs based on low-dimensional perovskites in addition to 3D perovskites. Fig. 5c shows the normalized EL spectra of these devices which exhibit a similar shift tendency to the PL spectra.

**Fig. 6:**
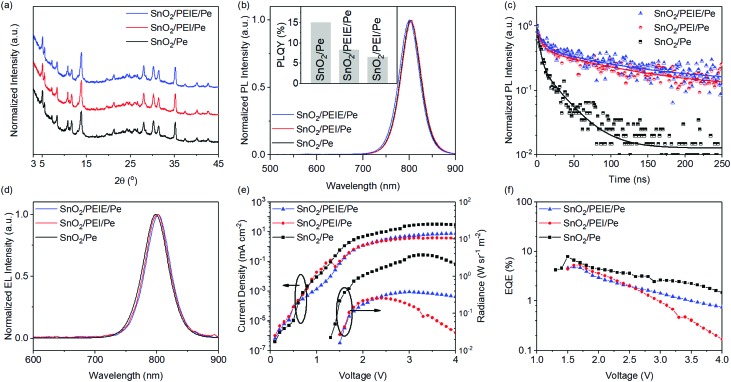
(a) XRD patterns, (b) PL spectra, (c) PL decay curves of the FAPbI_3_: 60 mol% NMAI films prepared on SnO_2_, SnO_2_/PEI, and SnO_2_/PEIE, respectively. (d) Normalized EL spectra, (e) current density–voltage–radiance curves and (f) EQE–voltage curves of the FAPbI_3_: 60 mol% NMAI based PeLED devices with SnO_2_, SnO_2_/PEI, and SnO_2_/PEIE as the ETLs.

It is reported that interfacial materials, such as PEIE and PEI, can modify the ETL surface and show favourable effects on the device performances.^11^,^20^ For instance, Wang *et al.* reported that 3D perovskites show a better morphology of PEI-modified ZnO, and consequently deliver higher device performances.^11^ In addition, these interfacial materials are able to tailor the work function of the contacts and can be used to control electron injection.^47^ Therefore, it is desired to study their effects on SnO_2_ based PeLEDs. Fig. 6a shows the XRD patterns of the FAPbI_3_: 60 mol% NMAI films prepared on SnO_2_, SnO_2_/PEI and SnO_2_/PEIE substrates, respectively. These films show similar XRD patterns, suggesting that these two interfacial materials have a negligible impact on the perovskite crystal structure. The CBM of the PEIE-modified SnO_2_ film is changed from –4.17 eV to –3.73 eV due to the shift of the WF (Fig. S6, ESI†). The more matching energy level with the perovskite layer (–3.88 eV) facilitates easier electron extraction from the perovskite layer.^48^ Although these perovskite films show similar PL spectra (Fig. 6b), the perovskite films prepared on SnO_2_/PEI and SnO_2_/PEIE exhibit gradually reduced PLQYs, indicating PL quenching caused by these interfacial materials. The transient PL decay characteristics of these perovskite films were investigated and are shown in Fig. 6c. The transient PL decay spectra are fitted with a bi-exponential decay function consisting of two time constants, suggesting a fast decay pathway and a slow decay pathway in these films. The fast decay pathway is associated with the quenching of free carriers in the perovskite domains caused by the substrate layers. As shown in Table S2 (ESI†), the fraction of the short decay time constants declines in the film with PEIE (or PEI). In consideration of the thick perovskite film (∼480 nm, shown in Fig. S7, ESI†), the slow decay pathway can be assigned to the PL decay of the residual free carriers that are far from the interfacial layer.

The interfacial materials are then employed in PeLED devices, the performance efficiencies of which are shown in Fig. 6d–f. Compared with the device without an interfacial layer, the device using PEIE (or PEI) shows reduced current densities and higher turn-on voltages, which may be due to the insulating nature of these materials. Unlike those based on ZnO, the device with PEIE (or PEI) modified SnO_2_ exhibits detrimental effects on the device performances.^11^,^15^ The PeLEDs with PEIE and PEI deliver EQEs of 4.9% and 5.4%, respectively, lower than that (7.9%) of the device without the interfacial layer. The results are in line with the PL measurements and confirm that the interfacial materials (PEIE and PEI) show detrimental effects due to increased non-radiative recombination. This observation may be useful for perovskite solar cells where charge extraction is found to be favourable.

## Conclusions

In conclusion, for the first time, we have systematically studied the effects of solution-processed SnO_2_ as the ETL in n–i–p structured PeLEDs based on both three- and low-dimensional perovskites. SnO_2_ exhibits a smooth morphology, suitable energy levels, good electron-transporting properties and excellent transparency with transmission values greater than 95% in ultraviolet–visible–infrared regions. In addition, the 3D perovskites (FAPbI_3_ and MAPbI_3_) show considerably enhanced chemical compatibility with SnO_2_ compared with ZnO. A series of PeLEDs are fabricated using SnO_2_ as the ETL, among which a high EQE of 7.9% is realized, demonstrating that SnO_2_ can be a good solution-processed ETL for n–i–p structured PeLEDs. Furthermore, interfacial studies suggest that the interfacial materials (PEI and PEIE) facilitate charge extraction in the SnO_2_ based perovskite films and consequently cause PL quenching and lower efficiencies.

## Conflicts of interest

There are no conflicts to declare.

## Supplementary Material

Supplementary information
