# Supplementary material for: Efficient perovskite light-emitting diodes based on a solution-processed tin dioxide electron transport layer
Source: J Mater Chem C Mater. 2018 Jun 6;6(26):6996–7002. doi: 10.1039/c8tc01871e (PMC6333275; doi:10.1039/c8tc01871e)
Supplement: Supplementary file 1 [file TC-006-C8TC01871E-s001.pdf]

Electronic Supplementary Material (ESI) for Journal of Materials Chemistry C.
This journal is © The Royal Society of Chemistry 2018

Electronic Supporting Information

Efficient Perovskite Light-Emitting Diodes Based on Solution-Processed Tin Dioxide Electron Transport Layer

Heyong Wang,[a] Hongling Yu,[a] Weidong Xu,[a] Zhongcheng Yuan,[a] Zhibo Yan,[ab] Chuanfei Wang,[a] Xianjie Liu,[a] Mats Fahlman,[a] Jun-Ming Liu,[b] Xiao-Ke Liu,*[acd] and Feng Gao*[a]

a. Department of Physics, Chemistry and Biology (IFM), Linköping University, Linköping 58183, Sweden.

b. Laboratory of Solid State Microstructures, Innovation Center of Advanced Microstructures, Nanjing University, Nanjing 210093, P. R. China.

c. State Key Laboratory of Luminescent Materials and Devices, South China University of Technology, Guangzhou 510640, P. R. China.

d. State Key Lab of Silicon Materials, Zhejiang University, Hangzhou 310027, P. R. China.

E-mail: xiaoke.liu@liu.se; feng.gao@liu.se

Experimental Section

Materials: The SnO_2 colloid precursor was obtained from Alfa Aesar (tin(IV) oxide, 15% in H_2O colloidal dispersion). The concentration of SnO_2 aqueous solution was diluted to be 5% before being used. Colloidal ZnO nanocrystals were synthesized following the reported solution-precipitation process.[11] FAI and MAI were obtained from Dyesol, PbI_2 was obtained from TCL (≥98.0% purity). NMAI was synthesized through the following procedures: hydroiodic acid (4.34 g, 45 wt% in water) was added to a stirring solution of 1-naphthalenemethylamine (12.72 mmol) and ethanol (30 ml), which was then kept at 0 ℃ for 2 h. The solution was then evaporated at 50 ℃ to give the NMAI precipitates, which was washed three times with ethyl acetate and then dried under vacuum. Other chemicals were obtained from Sigma-Aldrich.

The 3D perovskite precursor solutions were prepared by dissolving stoichiometric PbI_2, FAI, or MAI in DMF with a concentration of 0.7 M Pb^{2+}. The low-dimensional perovskite precursor solutions were prepared by adding NMAI into the $FAPbI_3$ precursor solutions with various molar ratios. All the solutions were stirred at 50 ℃ for 2 h before being used.

Preparation of thin films: The SnO_2 layer was prepared by spin-coating the dilute colloidal SnO_2 solution on ITO-coated glasses at 5000 rpm for 30 s, and then annealed in air at 150 °C for 15 min. Then the SnO_2 based substrates were transferred into a glovebox. PEIE and PEI were diluted in isopropyl alcohol to be 0.05 wt%, and then spin-coated at 5000 rpm for 30 s and annealed at 100 ℃ for 10 min in the glovebox.

For the 3D $FAPbI_3$ films, the precursor solutions were spin-coated on substrates at 4000 rpm for 45 s. During the spin-coating, 120 μL of chlorobenzene was poured on the as-synthesized wet film, and then annealed at various temperatures for 10 min. For the 3D $MAPbI_3$ films, the precursor solutions were spin-coated on substrates at 4000 rpm for 45 s and then annealed at various temperatures for 10 min. For low-dimensional perovskite films,

the resulting solutions were spin-coated on substrates at 2500 rpm for 30 s, followed by thermal annealing at 100 °C for 10 min.

Device fabrication and characterization: The SnO_2 and perovskite layers were prepared as the above-mentioned methods. The TFB layer was deposited from a chlorobenzene solution (10 mg/mL) at 3000 rpm for 30s. Then the films were transferred into a thermal evaporation system, where MoO_3 and Ag were deposited at the rates of 0.4 Å/s and 3.0 Å/s, respectively. The emitting area of the PeLEDs is 7.25 mm^2 defined by the overlapping area of the ITO pattern and the Ag electrode. Characterizations of the devices were carried out at room temperature in a nitrogen-filled glovebox. Specially, current density-voltage (J-V) characteristics were recorded by Keithley 2400 source meter. Forward-viewing spectral radiant flux was measured by an integrating sphere coupled with a QE65 Pro spectrometer. The EQE and radiance were calculated with the assumption that the PeLEDs are ideal Lambertian emitters, which are isotropic, emitting with equal radiance into any solid angle within the forward-viewing hemisphere.

Characterization: Atomic force microscopy (AFM) images were collected in a non-contact mode in air (Park XE7). The surface and cross-sectional SEM images were taken on a Philips XL30 FEG SEM operated at 3 keV. X-ray diffraction patterns were obtained using an X-ray diffractometer (Panalytical X'Pert Pro) with an X-ray tube (Cu Kα, λ = 1.5406 Å). UPS were collected in Scienta ESCA 200 spectrometer with a standard He-discharge lamp ($h\nu$ = 21.22 eV) with a total energy resolution of about 80 meV. The work function of film was extracted from the edge of the secondary electron cutoff of the UPS spectrum by applying a bias of -3 V to the sample. Absorption spectra were measured with a PerkinElmer model Lambda 900. Steady state PL spectra and PLQY were recorded with a 450 nm laser and an Andor spectrometer (Shamrock sr-303i-B, coupled to a Newton EMCCD detector) at room temperature in air. Time-correlated single photon counting (TCSPC) was performed on a

home-built setup, which contains diode lasers driven by a DH400, PicoQuant laser controller, a monochromator coupled with a micro channel plate photomultiplier tube (from Hamamatsu - R3809U-50) and TCSPC electronics (Lifespec-pc and VTC900 PC card from Edinburgh Instruments). A 407 nm laser generates pulses with full width at half maximum of 80 ps and repetition rates of 2.5 MHz.

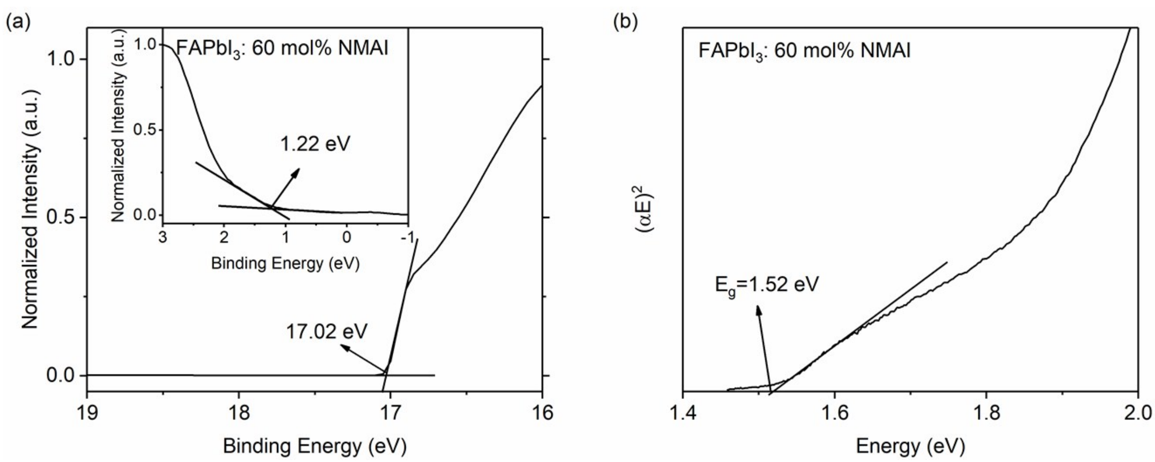

Fig. S1. (a) UPS spectra and (b) Tauc plot of $FAPbI_3$: 60 mol% NMAI film deposited on SnO_2.

The work function (WF) of $FAPbI_3$: 60 mol% NMAI film can be obtained by 17.02 eV – 21.2 eV = –4.18 eV. The valence band maximum (VBM) can be estimated by –4.18 eV – 1.22 eV = –5.40 eV. The conduction band minimum (CBM) can be calculated by $E_C = E_V + E_g$ = –5.40 eV + 1.52 eV = –3.88 eV. Similarly, the CBM and VBM of SnO_2 film are calculated to be –4.13 eV and –7.91 eV, respectively.

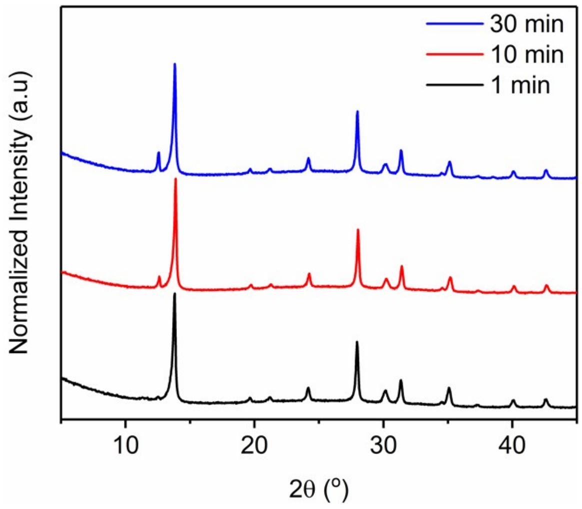

Fig. S2. XRD patterns of $FAPbI_3$ films deposited on SnO_2 films annealed at 160 ℃ with different annealing time.

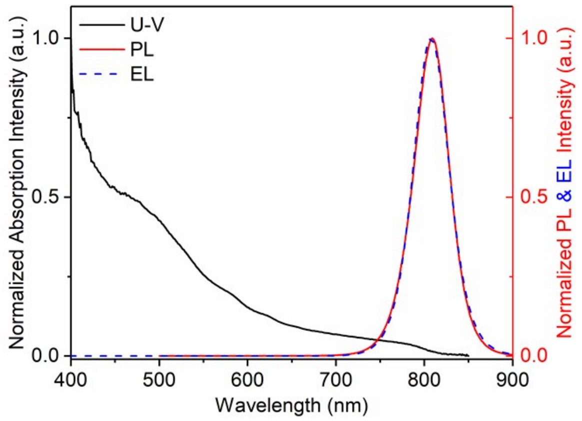

Fig. S3. Absorption, PL and EL spectra of the $FAPbI_3$ film deposited on SnO_2.

Table S1. XRD and PL parameters of the $FAPbI_3$: *x* mol% NMAI based films.

NMAI [mol%]	FWHM of XRD peak [°]	Crystalline size [nm]	PL peak [nm]	EL peak [nm]
0	0.28	28.3	811	810
20	0.36	21.9	795	801
40	0.38	20.8	800	794
60	0.48	16.5	798	799
80	0.49	16.2	763	788
100	0.43	18.4	731	755

The average crystalline size (*d*) of the films can be estimated by using Debye-Scherrer equation, which is expressed as

$$d=K\lambda/\beta\cos(\theta) \quad \text{(S1)},$$

where K (0.89) is a shape factor, λ is the X-ray wavelength (0.15406 nm), and β and θ are the values of full width at half maximum (FWHM) in radian and the Bragg angle of the (111) peak, respectively.

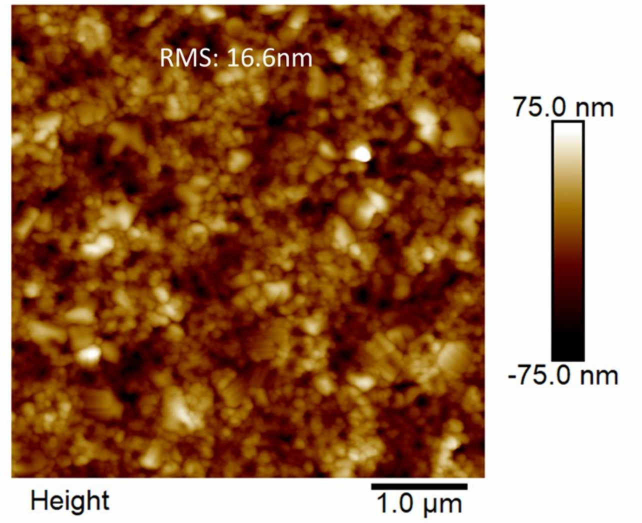

Fig. S4. AFM image of $FAPbI_3$: 60 mol% NMAI film deposited on SnO_2.

Table S2. Fitting parameters of transient PL decay plots.

	A_1 [%]	τ_1 [ns]	A_2 [%]	τ_2 [ns]	τ_{ave} [ns]
**SnO_2/Perovskite**	83.6	6.5	16.4	45	29
**SnO_2/PEIE/Perovskite**	58.6	9.3	41.4	125	114
**SnO_2/PEI/Perovskite**	54.2	11	45.8	117	107

The measured PL decay curves are fitted by using Equation S2. The average lifetime constants are calculated by using Equation S3 and S4.

$$decay_{(t)} = y_{(t_0)} + a_1 e^{-t/\tau_1} + a_2 e^{-t/\tau_2} \quad \text{(S2)},$$

$$\tau_{ave} = \frac{A_1\tau_1^2 + A_2\tau_2^2}{A_1\tau_1 + A_2\tau_2} \quad \text{(S3)},$$

$$A_1 = \frac{a_1}{a_1 + a_2}, \; A_2 = \frac{a_2}{a_1 + a_2} \quad \text{(S4)},$$

where A_1 and A_2 are coefficients, τ_1 and τ_2 are decay time constants.

.

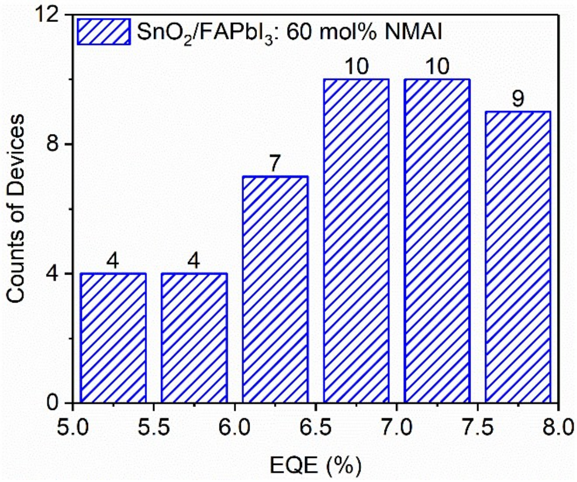

Fig. S5. Histogram of peak EQEs of 44 devices based on SnO_2/$FAPbI_3$: 60 mol% NMAI.

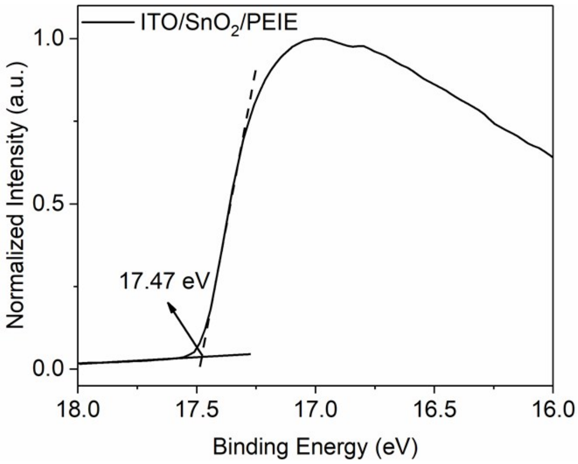

Fig. S6. UPS cutoff edge of the SnO_2/PEIE film.

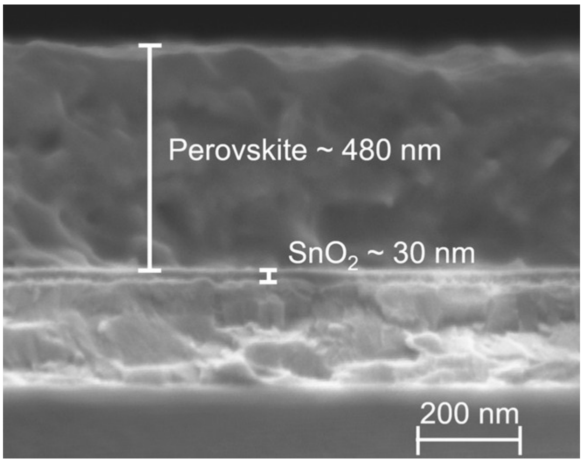

Fig. S7. Cross-sectional SEM image of $FAPbI_3$: 60 mol% NMAI film deposited on a glass/ITO/SnO_2 substrate.
